# Integrated Cantilever-Based Flow Sensors with Tunable Sensitivity for In-Line Monitoring of Flow Fluctuations in Microfluidic Systems

**DOI:** 10.3390/s140100229

**Published:** 2013-12-23

**Authors:** Nadine Noeth, Stephan Sylvest Keller, Anja Boisen

**Affiliations:** Department of Micro- and Nanotechnology, Technical University of Denmark, DTU Nanotech, Building 345E, DK-2800 Kgs. Lyngby, Denmark; E-Mails: nadinenoeth@googlemail.com (N.N.); anja.boisen@nanotech.dtu.dk (A.B.)

**Keywords:** micromechanical sensor, flow meter, pump characterization

## Abstract

For devices such as bio-/chemical sensors in microfluidic systems, flow fluctuations result in noise in the sensor output. Here, we demonstrate in-line monitoring of flow fluctuations with a cantilever-like sensor integrated in a microfluidic channel. The cantilevers are fabricated in different materials (SU-8 and SiN) and with different thicknesses. The integration of arrays of holes with different hole size and number of holes allows the modification of device sensitivity, theoretical detection limit and measurement range. For an average flow in the microliter range, the cantilever deflection is directly proportional to the flow rate fluctuations in the microfluidic channel. The SiN cantilevers show a detection limit below 1 nL/min and the thinnest SU-8 cantilevers a detection limit below 5 nL/min. Finally, the sensor is applied for in-line monitoring of flow fluctuations generated by external pumps connected to the microfluidic system.

## 1. Introduction

The progress in micro- and nanotechnology in the past years promoted the miniaturization of bio-/chemical sensors used for DNA analysis, pharmaceutical screening, medical diagnostics and environmental analysis [1,2]. For example, optical sensors such as optical waveguides or luminescence-based detection on chip [3] or micromechanical sensors [4] have been presented. In parallel, advanced microfluidic components such as pumps, mixers and valves have been developed [5]. This has allowed the integration of the sensors in microfluidic systems including pretreatment of the analyte and transport to the actual sensor surface [6]. The advantages of micro- and nanotechnology in these applications are reduced sample volume, faster response time during sample analysis and the possibility to detect low concentrations of analyte.

For continuous measurement of analyte concentrations on such a sensor platform, a stable liquid flow in the microfluidic system is important. Flow fluctuations are directly transformed into noise in the output signal of the bio-/chemical sensor. Therefore, it is important to monitor the flow in microfluidic channels. In-line measurement of flow fluctuations in the microfluidic system with an integrated flow sensor allows the characterization of the fluidic noise. As a consequence, the output of the bio-/chemical sensor can be corrected, particularly if the noise is periodic.

Due to the small sample volumes and the low flow rates a flow sensor with a high sensitivity is required. Various types of flow sensors with potential for integration in microfluidic systems have been proposed [5,7], such as thermal anemometers [8–12], optical devices [13–14], pressure-based sensors [15–18] or sensors based on electrical admittance [19–21]. Recently, cantilever-based flow meters have been presented. Typically, the cantilevers are positioned perpendicularly to the flow in a microfluidic channel [22–25]. In this configuration, the bending of the devices increases at higher flow rate due to an increase in pressure on the surface of the cantilevers. Alternatively, pre-stressed cantilevers are placed at a small angle to the walls of the flow channel and the change of bending due to a change in shear stress is measured [26]. The measurement of the beam deflection is done with optical or piezoresistive read-out. These flow sensors show an extremely short response time and the deflection is seen to be directly proportional to the flow rate in the microfluidic system. The sensitivity of cantilever-based flow sensors is potentially increased by decreasing the stiffness of the cantilever. This is achieved selecting a beam material with a low Young's modulus, such as SU-8 [22] or polydimethylsiloxane (PDMS) [26] or minimizing the thickness of the cantilever. The devices presented in literature show detection limits in the order of μL/min.

We have previously reported the fabrication of a flow sensor consisting of a 3.7 μm thick SU-8 cantilever positioned perpendicularly to the flow direction inside a microfluidic channel [27]. The beam deflection is measured with an optical read-out system. A laser is focused on a metal pad at the apex of the cantilever. The reflection of the laser beam is detected by a position sensitive photo-diode (PSD). An array of holes is integrated in the cantilever surface, which results in a liquid flow through the cantilever. With the initial sensor design a detection limit of a few nL/min has been achieved.

The integration of holes in the cantilever surface is expected to have two competing effects: on the one hand, the flow resistance of the cantilever decreases for a larger number of holes, which should result in lower bending at equal flow rate. On the other hand, the mechanical stiffness of the beam is reduced upon integration of holes which should result in higher deflection at equal flow rate. Due to the device geometry and the integration of the perforated cantilever into a microfluidic channel, it has been challenging to analytically predict or to simulate the behaviour of the proposed sensor. Here, we present a detailed experimental investigation of the influence of cantilever material, thickness and design on sensitivity, detection limit and measurement range of the described flow sensors. For this purpose, SiN and SU-8 devices with variable thicknesses and designs are fabricated and characterized. Finally, we demonstrate the ability to perform in-line monitoring of flow fluctuations in a microfluidic system using the integrated cantilever-like sensor. For this purpose, the flow generated by four different microfluidic pumps is compared.

## 2. Experimental Section

### 2.1. Device Designs

**Figure 1.**
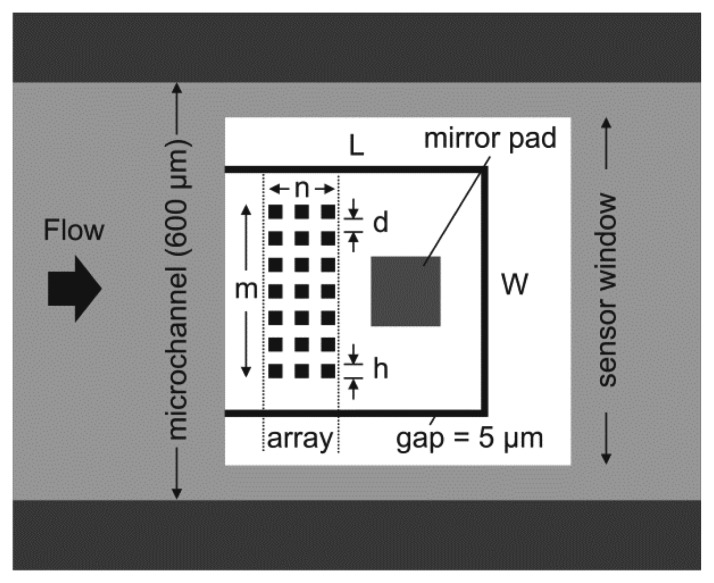
Design parameters for cantilever-based flow sensor integrated in a microfluidic channel; *W* = cantilever width, *L* = cantilever length, *h* = hole size, *d* = distance between holes, *n* = columns in array, *m* = rows in array.

Cantilever-based flow sensors with beam length *L* = 370 μm and width *W* = 340 μm are fabricated in SU-8, a negative epoxy-based photoresist, and SiN. SU-8 cantilevers with three different thicknesses of 3.7, 5.4 and 11.2 μm are fabricated. The SiN cantilevers have a thickness of 580 nm. Arrays of quadratic holes with different designs are integrated in the cantilevers in the area between 50–200 μm from the beam clamping as shown in Figure 1. The dimensions of the hole array (columns *n*, rows *m*), the hole size *h*, the distance between the holes *d* and as a consequence the number of holes *N* = *n* × *m* are varied to investigate the influence of the sensor design on the device sensitivity. Table 1 summarizes the design parameters and materials used in the experiments.

The different designs are further referred to as design “*h*(*N*)”, e.g., “10(180)” for a sensor with 180 holes with a size of 10 × 10 μm^2^. The porosity *p* of the different designs is calculated as the ratio between the total area of the holes *A_h_* and the total cantilever area *A_c_*:

(1) $$p=\frac{A_{h}}{A_{c}}=\frac{nmh^{2}}{LW}$$

The width of the gap between the microchannel and the cantilever is 5 μm for all the sensor designs.

### 2.2. Fabrication of SU-8 Devices

**Figure 2.**
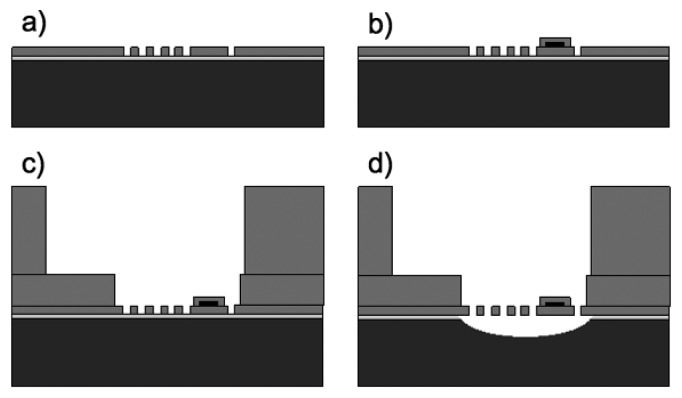
Fabrication sequence for the SU-8 devices: (**a**) Patterning of perforated cantilevers in SU-8. (**b**) Patterning of Al pad at the apex of the cantilever and encapsulation with SU-8. (**c**) Patterning of microfluidic channel in SU-8. (**d**) Isotropic etch of the Si support substrate.

**Figure 3.**
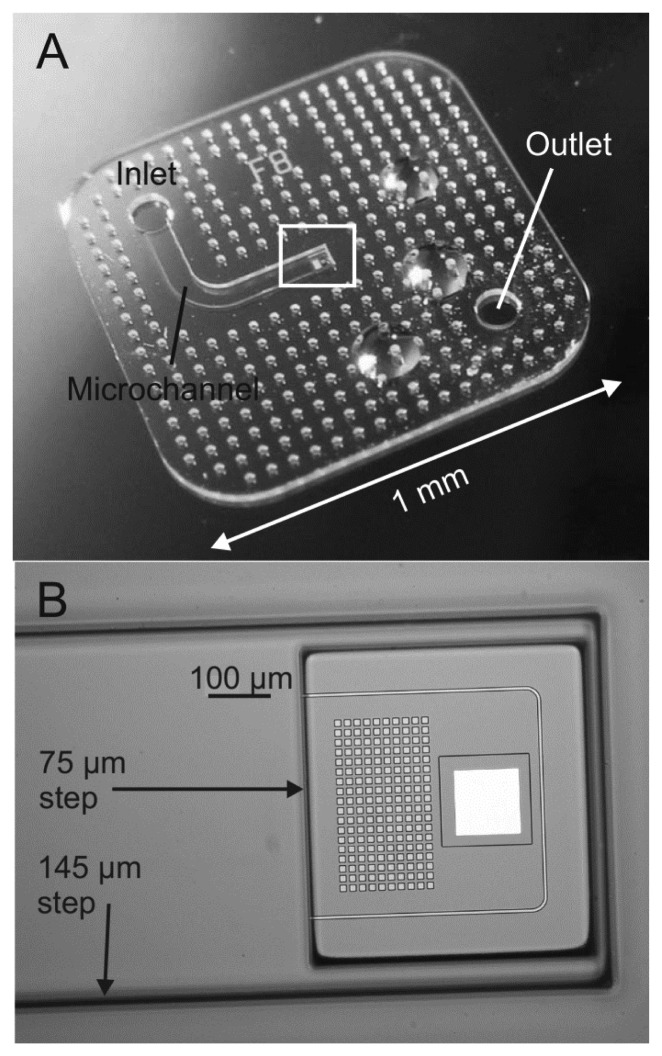
(**a**) SU-8 microchip with microfluidic channel; (**b**) top view of a 3.7 μm thick SU-8 flow sensor containing 180 holes with dimensions 10 × 10 μm^2^.

The fabrication of the SU-8 cantilevers is based on a process described earlier and the main steps are illustrated in Figure 2 [27]. The first processing step is the deposition of a fluorocarbon anti-stiction coating onto a Si substrate allowing release of the SU-8 chips by tweezers after completed fabrication [28]. The perforated cantilevers are defined in a first layer of SU-8 by standard UV photolithography (Figure 1a). SU-8 2005 (Microchem, Newton, MA, USA) is spin coated for 30 s onto the substrate with parameters depending on the thickness of the cantilevers according to Table 2. The thickness of the SU-8 layers is measured with a profilometer with a wafer scale uniformity better than 50 nm for the 3.7/5.4 μm thick films and 100 nm for the 11.2 μm thick film. It has earlier been shown that incomplete removal of the solvent before UV exposure and a post exposure bake (PEB) at low temperature result in highly cross-linked SU-8 films with low residual stress [29]. Therefore, the soft bake for the 3.7 μm and 5.4 μm thick SU-8 layers is replaced by solvent evaporation for 3 h at ambient temperature (20 °C). For the 11.2 μm thick SU-8 film, a short soft bake for 30 min at 50 °C is required to avoid stiction between substrate and mask after UV exposure in hard contact mode. The exposure dose for the different film thicknesses has been optimized. High exposure dose results in low residual stress but affects the lithographic resolution [29]. For 3.7 μm and 5.4 μm thick SU-8 films, the optimal exposure dose is 200 mJ/cm^2^. For 11.2 μm thick SU-8 cantilevers the dose has to be reduced to 150 mJ/cm^2^ to definition of holes with *h* = 5 μm. A PEB for 1 h at 50 °C is followed by development in propylene glycol methyl ether acetate (PGMEA) and rinsing in 2-propanol (IPA). A second UV exposure with a dose of 500 mJ/cm^2^ ensures a high cross-linking of the SU-8 and a subsequent hardbake for 10 h at 120 °C reduces stress gradients in the SU-8 cantilevers [30]. With the optimized exposure parameters holes with dimensions as small as 5 × 5 μm^2^ are precisely transferred into 3.7 μm and 5.4 μm thick SU-8 layers. In 11.2 μm thick SU-8 layers a slight loss of resolution is observed and the smallest nominal mask dimensions of 5 × 5 μm^2^ result in holes of 4 × 4 μm^2^. As a consequence, the effective porosity for the design 5(405) in Table 1 is reduced to *p* = 5.2%. A 50 nm thick Al pad is structured at the apex of the cantilevers to achieve the reflectivity required for optical read-out. A passivation layer of 3.7 μm thick SU-8 is structured on top of the pad to protect the Al from delamination during experiments (Figure 2b). This is followed by subsequent structuring of two SU-8 layers with a thickness of 75 μm and 145 μm respectively, as described in detail in [27]. The first one defines the bottom of the microfluidic channel which includes a window above the perforated cantilever and the second one the body chip and the microfluidic inlet channel (Figure 2c). An isotropic Si etch in a deep reactive ion etch (DRIE) equipment underetches the perforated cantilevers to obtain free-standing structures. After a final hardbake at 120 °C for 2 h the SU-8 body chip shows minimal adhesion to the substrate and can be picked up by tweezers (Figure 2d). Figure 3 shows a released SU-8 flow sensor.

### 2.3. Fabrication of SiN Devices

**Figure 4.**
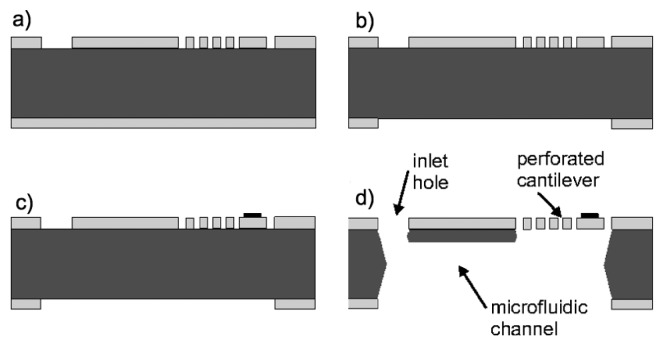
Fabrication sequence for the SiN devices: (**a**) Patterning of perforated cantilevers in the SiN layer on the front side of the wafer. (**b**) Patterning of etch mask for microfluidic channel in the SiN layer on the back side of the wafer. (**c**) Patterning of metal pad at apex of the cantilever. (**d**) Definition of microchannel and release of the SiN devices in KOH.

**Figure 5.**
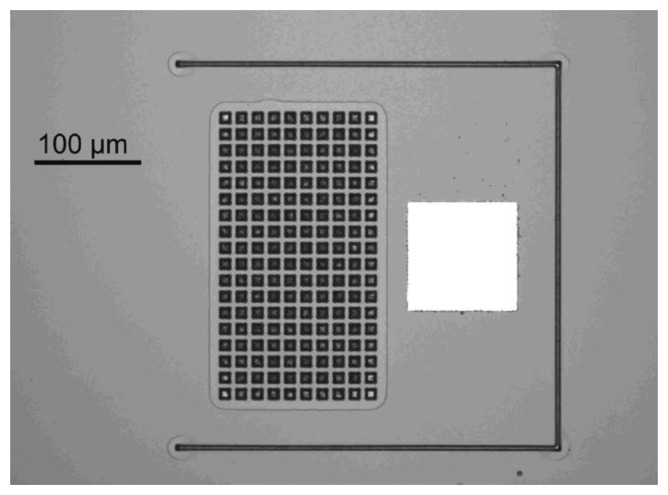
Top view of a 580 nm thick SiN flow sensor containing 180 holes with dimensions 10 × 10 μm^2^.

A fabrication process similar to the one reported by van Rijn *et al.* is used to fabricate the SiN cantilever sensors as illustrated in Figure 4 [31,32]. A 580 nm thin layer of Si rich (low stress) nitride (stress 350 MPa) is deposited on both sides of a 525 μm thick double side polished Si substrate via a LPCVD process. A standard lithography process is carried out on the front and back side of the Si wafer to structure the SiN layers. Then, the SiN layers are etched by reactive ion etch (RIE) (Figure 4a,b). A 5 nm Cr/50 nm Au metal pad is deposited at the apex of the cantilever to achieve the reflectivity required for the optical read-out of the cantilever bending (Figure 4c). The SiN cantilevers are released in a KOH wet etch (28 wt.%, 6 h at 75 °C). The wafer is etched from both sides as seen in Figure 4d and a microfluidic channel is defined. Figure 5 shows an optical microscope image of a released SiN flow sensor.

### 2.4. Measurement Setup

**Figure 6.**
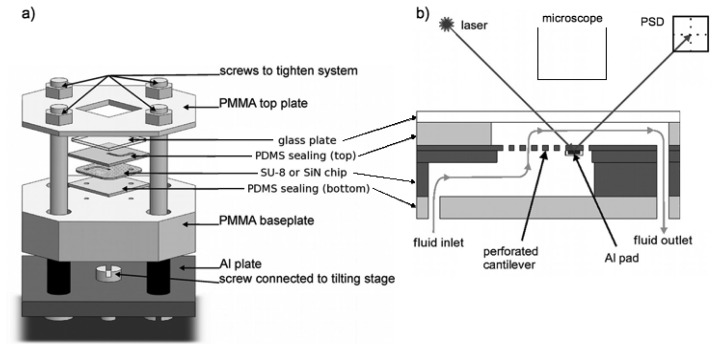
Schematics of the mechanical holder with the microfluidic system and the SU-8 or SiN chip: (**a**) Three-dimensional view of the mechanical holder. (**b**) Side view of the microfluidic system; the grey line with the arrows indicates the direction of the fluid flow.

The measurement setup consists of four main parts: The SU-8 or SiN chip, a mechanical holder with integrated microfluidic system, an optical read-out system and a fluid handling system [27]. The cantilever chip (SU-8 or SiN) is inserted in a microfluidic system which is clamped in a mechanical holder as shown in Figure 6a to avoid leakage. The microfluidic inlet channel in the cantilever chip is sealed with a bottom polydimethylsiloxane (PDMS) layer and in a top PDMS layer the microfluidic outlet channel is structured and sealed with a glass plate (Figure 6b). For the optical read-out, a laser is focused on the metal pad of the cantilever and the reflected beam is detected with a position sensitive photo-diode (PSD). A microscope objective is positioned above the cantilever to allow alignment of the laser.

### 2.5. Flow Sensor Characterization

A self-built gravity pump, consisting of a blue cap glass bottle from VWR International LLC (Radnor, PA, USA) positioned above the measurement setup, is connected to the inlet channel of the mechanical holder via microfluidic tubing (FEP NAT 1520, IDEX Health & Science LLC, Oak Harbor, WA, USA). The flow rate of the gravity pump is controlled with a mechanical valve P445 from Upchurch Scientific LLC (Oak Harbor, WA, USA) and an external thermal flow meter SLG1430-480 from Sensirion (Stäfa, Switzerland) connected to the microfluidic outlet channel. At the same time, the Sensirion flow meter is used as a reference.

All experiments are carried out in Milli-Q water, which is initially deoxygenated for 2 h in a vacuum chamber. The system is flushed with Milli-Q water before the actual cantilever deflection measurement to ensure that no air bubbles are trapped in the microfluidic channel. Then, a constant flow of 10 μL/min for the SU-8 devices and 2 μL/min for the SiN devices is applied. The flow for the SiN devices is lower compared to the SU-8 devices because a flow of 10 μL/min is outside the measurement range (see Section 3.2). Next, the laser of the optical read-out system is aligned to the metal pad at the apex of the cantilever and the reflected laser beam is focused on the center of the PSD. The alignment of the laser is done during an applied flow, which means that the measured cantilever deflection is relative to the initial bending caused by the initial flow. After laser alignment, the flow rate is increased step by step with the mechanical valve and the output of the commercial flow meter and the perforated cantilever is recorded. All the initial experiments are carried out at low flow rates (0–40 μL/min), where the bending of the cantilevers is small and the laser beam is reflected on the PSD without re-alignment.

### 2.6. Flow Monitoring

The flow sensor with an 11.2 μm thick SU-8 cantilever with 180 holes of 10 × 10 μm^2^ (design 10(180) in Table 1) is employed to demonstrate the potential of the fabricated sensors for flow monitoring. Four different pumps are connected to the devices and the generated flow is analyzed. The PhD 22/2000 syringe pump from Harvard Apparatus (Holliston, MA, USA) has a working range of 0.1 nL/min to 221 mL/min and two outlet channels. The syringe pump 22 from Harvard Apparatus works at low flow rates from 33.3 pL/min to 55.1 mL/min and also has two outlet channels. The peristaltic pump VS4-10R-MIDI from Watson Marlo Alitea (Stockholm, Sweden) operates at flow rates ranging from 0.5 μL/min–17.5 mL/min and has four outlet channels. The flow output from the syringe pumps and the peristaltic pump is controlled by an integrated microprocessor. Finally, the self-built gravity pump described above is examined. Both syringe pumps and the gravity pump are set to a flow rate of 30 μL/min and the peristaltic pump to 10 μL/min. The relative cantilever deflection during 10 min is compared to the flow rate measured with the commercial flow sensor from Sensirion.

## 3. Results and Discussion

### 3.1. Comparison of Fabrication Processes

For the fabrication of the SU-8 sensors, five lithographic masks are used and six days of processing are required. Optimization of several steps of the SU-8 fabrication process has been necessary, since the fabrication of cantilever devices is less established for SU-8 than for SiN. The SiN fabrication process requires only three lithographic masks and three days of processing. The SiN fabrication process is faster and less complex than the SU-8 process, but since more expensive machines are used during fabrication, it is more costly.

Both fabrication processes have their limitations for the range of thicknesses of cantilever-based flow sensors that can be fabricated. The SU-8 fabrication process is restricted by two factors: (1) For SU-8 layers thinner than 3 μm release of the flow sensors from the substrate is impossible. (2) For SU-8 layers thicker than 10 μm the lithographic resolution is decreasing. For SiN sensors the fabrication process is mainly limited by the intrinsic stress in the SiN after deposition, which increases with increasing thickness of the SiN layer. Fabrication of flow sensors with a thickness of 1 μm failed due to that reason.

### 3.2. Relative Cantilever Deflection

**Figure 7.**
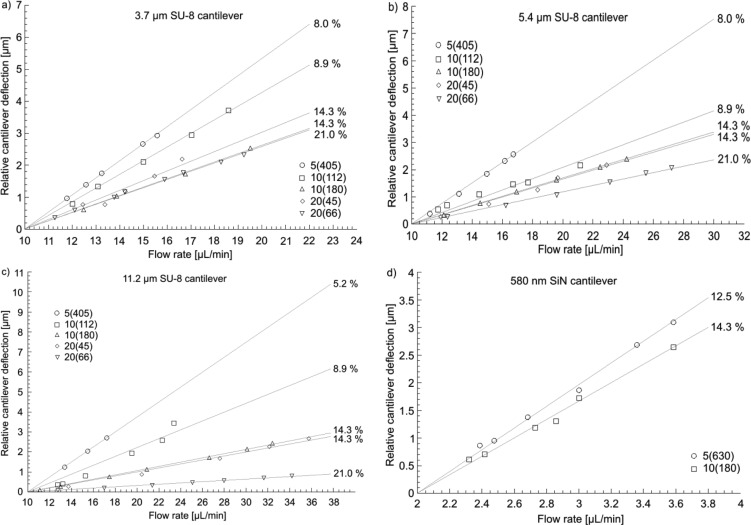
Relative cantilever deflection as a function of flow rate for various sensor designs: (**a**) 3.7 μm thick SU-8 cantilevers. (**b**) 5.4 μm thick SU-8 cantilevers. (**c**) 11.2 μm thick SU-8 cantilevers. (**d**) 580 nm thick SiN cantilevers. The porosity *p* for the corresponding design is indicated (%). For design 5(405) in (c), p = 5.2% due reduced lithographic resolution for 11.2 μm thick SU-8.

Figure 7 shows relative cantilever deflections at variable flow rate for devices with different porosity and thickness. For all devices, the cantilever bending increases with increasing flow rate due to increasing pressure on the cantilever surface exposed to the flow. The lines indicate that there is a linear relationship between the flow rate and the cantilever deflection in the selected range of flow rates independent of device material and geometry. Figure 7a–c demonstrates that thicker SU-8 cantilevers respond less to identical changes of flow rate. For the SiN cantilevers, a change of flow rate of 2 μL/min results in deflections of 3 μm (Figure 7d), which is considerably more than for the SU-8 cantilevers. Furthermore, the relative cantilever deflection at identical flow rate decreases with increasing porosity. Remarkably, identical porosity (*p* = 14.3%) results in the same deflection curve in Figure 7b and Figure 7c, even though the sensor design is different (180 holes with 10 × 10 μm^2^ and 45 holes with 20 × 20 μm^2^).

In principle, the mechanical stiffness of the cantilevers is reduced upon integration of holes, which should result in higher deflection for higher porosity. However, at the same time the flow resistance of a cantilever with a larger number of holes is reduced, which results in lower bending for higher porosity. The deflection curves in Figure 7 demonstrate that the decrease of flow resistance upon introduction of holes has a more important effect on the bending of the cantilever-based flow sensors than the decrease of mechanical stiffness.

### 3.3. Device Sensitivity and Detection Limit

The sensitivity *S* of the different flow sensors corresponds to the slope of the deflection curve in Figure 7. The linearity indicates that the device sensitivity is constant in the observed range of flow rates. Deflection measurements such as the ones shown in Figure 7 are repeated at least three times for each device design. Finally, the average slope of the deflection curves is calculated and defined as the sensitivity *S* of the flow sensors in the investigated range of flow rates.

**Figure 8.**
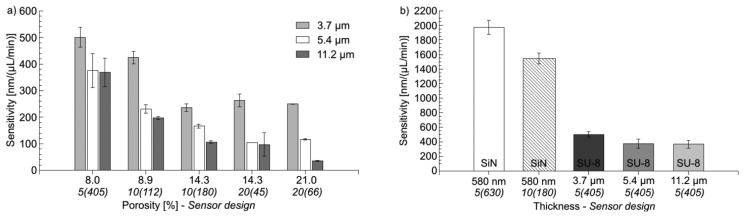
(**a**) Sensitivity of SU-8 flow sensors with variable thickness and porosity/hole design. (**b**) Comparison of sensitivity of SiN cantilevers and most sensitive SU-8 cantilevers. For design 5(405), p = 5.2% due reduced lithographic resolution for 11.2 μm thick SU-8.

Figure 8a shows that the sensitivity of SU-8 cantilevers depends on thickness and sensor design. An increase in thickness results in less sensitive devices. This is explained with the higher stiffness of the cantilevers due to a higher spring constant. Furthermore, for devices with *p* ≤ 14.3%, the sensitivity decreases considerably with increasing porosity, where the sensitivity for designs with *p* ≥ 14.3% remains more or less constant. Here, two competing effects have to be considered. It could be expected that increasing porosity results in a lower stiffness of the cantilever and higher sensitivity. However, the integration of holes allows the liquid to partially flow through the cantilever and as a consequence the flow resistance of the devices decreases, which results in a decrease of the sensitivity for increasing porosity. The experiments indicate that the flow resistance of the fabricated cantilevers rapidly decreases upon the introduction of holes, which has a dominating effect on the sensitivity compared to the simultaneous decrease of mechanical stiffness. Figure 8b shows a comparison of the sensitivity of the most sensitive devices for each SU-8 thickness and the SiN cantilevers.

The most sensitive SU-8 cantilevers have 405 holes with nominal dimensions of 5 × 5 μm^2^ (design 5(405) in Table 1). The sensitivity of the SiN devices is more than twice the one of the most sensitive SU-8 cantilever (thickness = 3.7 μm). This is the case although SiN has a much higher Young's modulus (260 GPa, [33]) compared to SU-8 (4 GPa, [34]). The explanation is the much lower device thickness (580 nm), which reduces the stiffness of the cantilevers and compensates for the increase in Young's modulus.

The detection limit *R* = *R_opt_/S* of each flow sensors is determined with *R_opt_* = 1.5 nm being the detection limit of the optical read-out system. The results are summarized in Table 3. The SU-8 flow sensors show a detection limit between 3 nL/min and 43 nL/min depending on the thickness and the design. With the SiN perforated cantilevers a sub nL/min detection limit is achieved.

### 3.4. Measurement Range

The measurement range of the flow sensors is mainly limited by the method for optical read-out. Cantilever deflections >±3 μm are too large to allow reflection of the laser beam on the PSD. For the experiments in Figure 7, the measurement range of the 580 nm thick SiN cantilever is limited to a few μL/min. Compared to that the least sensitive SU-8 cantilever (design 20(66) in Figure 7c), only deflects 800 nm for a flow rate increase of 30 μL/min for an initial flow rate of 10 μL/min, which results in a measurement range of around ±100 μL/min.

The addition of a tilting stage (±5°) to the measurement setup allows refocusing of the laser beam onto the center of the PSD. With this approach measurements at higher flow rates are possible and approximately a six-fold increase of the measurement range of the flow sensors is achieved. The measurement range for the most sensitive SiN flow meter is expanded to ±12 μL/min. The most sensitive SU-8 cantilever (thickness = 3.7 μm, design 5(405)) has a larger range of ±80 μL/min. With the 11.2 μm thick SU-8 cantilevers with 66 holes of 20 × 20 μm^2^, which are the devices with the lowest sensitivity, flows of up to ±600 μL/min have been measured.

### 3.5. Device Sensitivity at High Flow Rate

For the measurements in Figure 7, a linear response of the cantilever deflection to changes of flow rate is observed and the sensitivity of the devices in the measurement range is constant. The integration of the tilting stage allows the analysis of the device sensitivity at higher flow rates. Here, the flow rate is increased to a higher initial value and the laser beam is refocused on the PSD using the tilting stage to compensate for higher initial bending of the cantilever. Then experiments and data analysis are repeated as described above.

**Figure 9.**
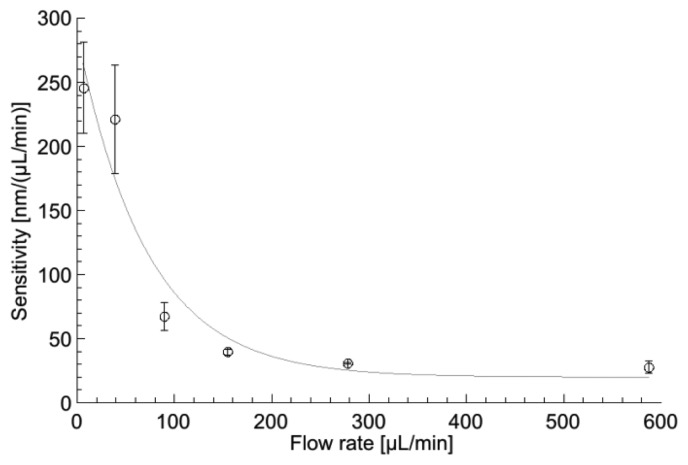
Sensitivity of the 11.2 μm thick SU-8 flow sensor with 112 holes of 10 × 10 μm^2^ at variable flow rate.

The experiments demonstrate that the sensitivity of the flow meter is not constant over the entire measurement range. Figure 9 shows the results obtained with a SU-8 cantilever with a thickness of 11.2 μm and 112 holes of 10 × 10 μm^2^. The highest sensitivity of the flow meter is achieved at low flow rate (<20 μL/min) and it decreases exponentially with increasing flow rate. At high flow rates, the gap between the cantilever and the microfluidic channel wall increases due to the large deflection of the beam. Therefore, an increasing amount of fluid passes the flow meter without interaction with the sensor surface, which results in lower sensitivity. Furthermore, the perpendicular load from the applied pressure onto the cantilever surface is reduced at larger bending, as it is partially converted into shear force. Nevertheless, the detection limit of the flow sensor in Figure 9 at a flow rate of 600 μL/min is still around 0.1 μL/min.

### 3.6. Flow Monitoring

**Figure 10.**
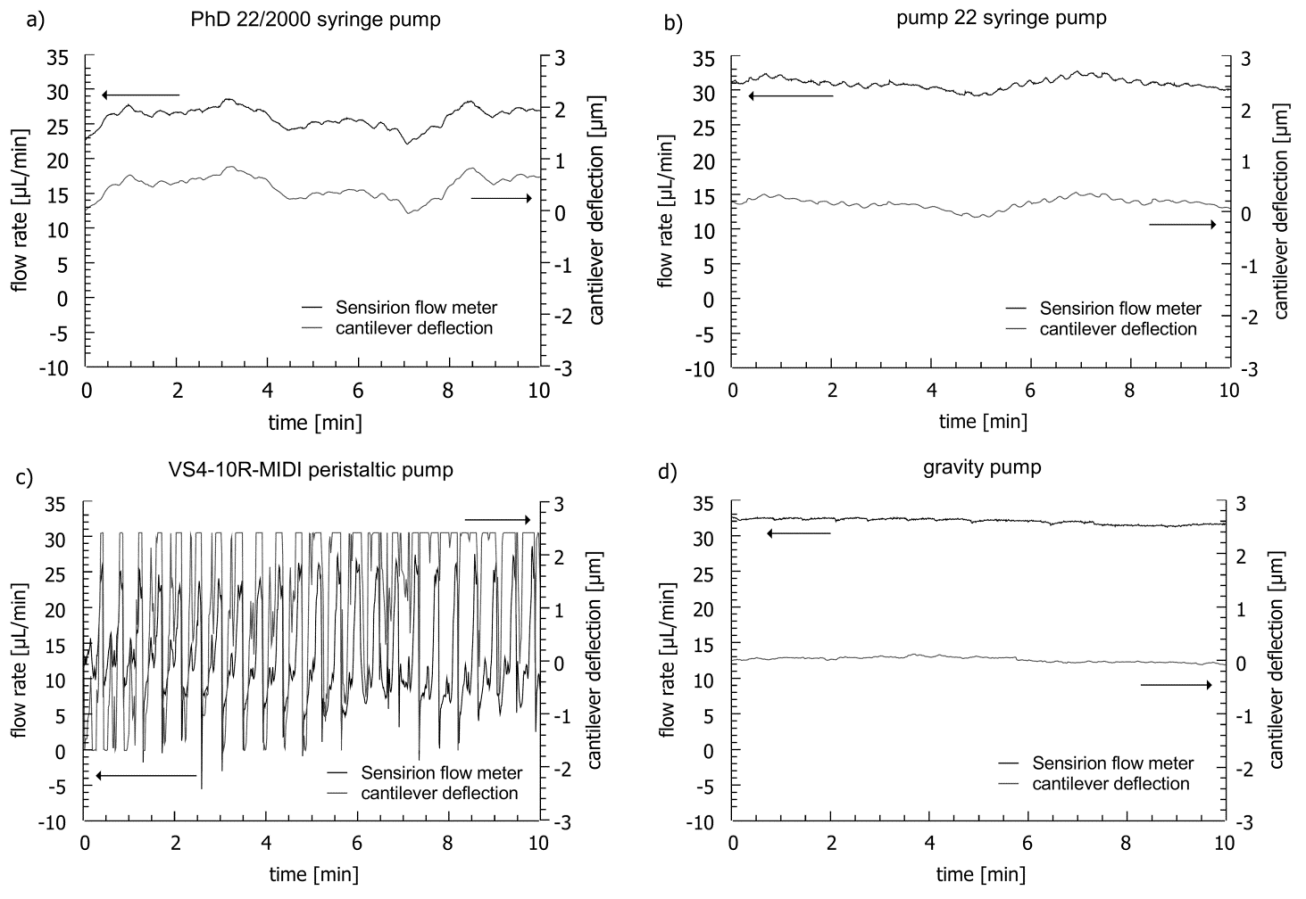
Flow stability measurement with the 11.2 μm thick SU-8 flow sensor with 180 holes of 10 × 10 μm^2^ and the thermal flow meter from Sensirion with four different pumps.

Figure 10 shows a comparison of the measured deflection of a cantilever-based flow sensor and the flow rate recorded with the commercial flow meter during 10 min for four different pumps. The bending response of the cantilever-based device concurs precisely with the flow rate measured with the reference for all experiments. The flow profiles of the four pumps are different. With the syringe pumps (PhD 22/2000 and 22) connected to the microfluidic system, a variation of the cantilever bending of ±465 nm and ±241 nm is measured respectively (Figure 10a,b). The measurements with the commercial sensor show that this corresponds to flow rate variations of ±3.3 μL/min (syringe pump PhD 22/2000) and ±1.8 μL/min (syringe pump 22). For the peristaltic pump, measurements at flow rates >10 μL/min have been impossible. The flow rate fluctuations generated by the peristaltic pump (±17.1 μL/min) are significant and the cantilevers deflect out of range of the optical readout (> ±3 μm) (Figure 10c). The gravity pump shows high flow stability and induces a deflection noise for the perforated cantilevers of less than ±106 nm corresponding to flow rate fluctuations of ±0.7 μL/min (Figure 10d).

**Figure 11.**
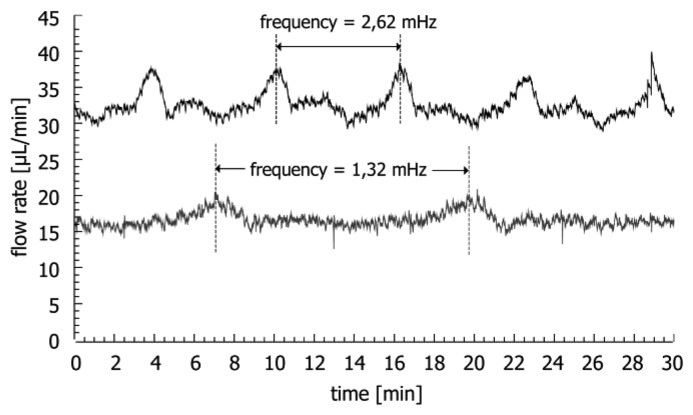
Flow rate measurement for PhD 22/2000 syringe pump (upper curve: flow rate 30 μL/min, lower curve: flow rate 15 μL/min).

The fluctuations observed for the peristaltic pump are caused by ten mechanical rollers, which press the liquid through the tubing. The two syringe pumps induce flow rate fluctuations which are periodic. Figure 11 shows the flow rate attained by the PhD 22/2000 syringe pump at average flow rates of 30 μL/min and 15 μL/min.

The frequency of the large periodic noise in the system is proportional to the flow rate. This implies that the noise is generated by the rotating actuator screws of the pump, which apply pressure on the clamped syringes. The in-line measurement might allow for characterization of the periodicity of the fluctuations at a specific flow rate and the correction of the sensor output. Furthermore, the syringe pump induces minor high frequency flow fluctuations probably due to vibrations generated by the screw movement. The amplitude of the noise from the gravity pump is very low. There, the calculated variation is mainly a result of the linear drift caused by the decreasing water level in the bottle during the experiments.

## 4. Conclusions

A highly sensitive flow sensor based on cantilever technology is presented. The cantilevers are fabricated in SU-8 and SiN. The influence of device design (hole size, porosity, thickness) and material (SU-8 and SiN) on device performance is discussed. The sensitivity of the devices decreases for a higher thickness and a larger porosity of the cantilever. For cantilever deflections below ±3 μm, the change of bending is directly proportional to changes in flow rate and the sensitivity is constant in this measurement range. The 580 nm thin SiN cantilevers have the highest device sensitivity and with that the lowest detection limit of below 1 nL/min. Due to the high sensitivity the measurement range is reduced to a few μL/min. The most sensitive SU-8 cantilevers have a detection limit of below 5 nL/min. With the least sensitive SU-8 devices (detection limit 42 nL/min) a measurement range as large as ±100 μL/min is achieved. Integration of a tilting stage (±5°) allows compensation for initial bending of the cantilevers and a six-fold expansion of the measurement range. However, the device sensitivity is not constant for the expanded measurement range and decreases at higher flow rates.

The characterization of microfluidic flow generated by four different pumps demonstrates that variations of flow rate in a microchannel are perfectly reflected in the response of the cantilever-like flow sensors. This study demonstrates the importance of in-line monitoring of flow fluctuations in microfluidic experiments. Assuming that a nano- or micromechanical sensor is placed in the microfluidic system and used for bio/chemical detection [4], the flow noise generated by external components such as pumps will significantly influence its performance. Particularly for periodic noise or drift, the in-line monitoring should open up for an analytical correction of the output of bio-/chemical sensors in the microfluidic system.

The developed flow sensors show very promising characteristics and a broad range of flow rates are covered with the different sensor designs, thicknesses and materials. A drawback of the sensor is the fact, that impurities in biological samples might result in clogging of the holes and thereby introduce noise in the sensor readout.

## Figures and Tables

**Table 1.** Design parameters and materials of the fabricated flow sensors; *h* = hole size, *d* = distance between holes, *n* = columns in array, *m* = rows in array, N = number of holes, p = porosity.

**Design h(N)**	**h (μm)**	**d (μm)**	**n**	**m**	**N**	**p (%)**	**Materials**
5(405)	5	5	15	27	405	8.0	SU-8
5(630)	5	3	18	35	630	12.5	SiN
10(112)	10	10	8	14	112	8.9	SU-8
10(180)	10	5	10	18	180	14.3	SU-8, SiN
20(45)	20	10	5	9	45	14.3	SU-8
20(66)	20	5	6	11	66	21.0	SU-8

**Table 2.** Spin coating parameters for the first SU-8 layer depending on the cantilever thickness.

**Thickness of SU-8 Cantilever**	**3.7 μm**	**5.4 μm**	**11.2 μm**
Spin coating speed	4,000 rpm	2,000 rpm	500 rpm
Spin coating acceleration	5,000 rpm s^−1^	5,000 rpm s^−1^	200 rpm s^−1^

**Table 3.** Flow rate detection limit (nL/min) of the developed flow meters depending on material, thickness and sensor design.

**Hole Design**	**Porosity (%)**	**SiN 580 nm**	**SU-8 3.7 μm**	**SU-8 5.4 μm**	**SU-8 11.2 μm**
5(405)	8.0		3.00	4.07	4.12^a^
5(630)	12.5	0.76			
10(112)	8.9		3.54	6.50	7.62
10(180)	14.3	0.97	6.36	9.03	14.16
20(45)	14.3		5.72	14.36	18.12
20(66)	21.0		6.00	12.91	42.20

a Porosity 5.2% due to lower lithographic resolution.
